# A Layered Searchable Encryption Scheme with Functional Components Independent of Encryption Methods

**DOI:** 10.1155/2014/153791

**Published:** 2014-02-25

**Authors:** Guangchun Luo, Ningduo Peng, Ke Qin, Aiguo Chen

**Affiliations:** School of Computer Science and Engineering, University of Electronic Science and Technology of China, Chengdu, Sichuan 611731, China

## Abstract

Searchable encryption technique enables the users to securely store and search their documents over the remote semitrusted server, which is especially suitable for protecting sensitive data in the cloud. However, various settings (based on symmetric or asymmetric encryption) and functionalities (ranked keyword query, range query, phrase query, etc.) are often realized by different methods with different searchable structures that are generally not compatible with each other, which limits the scope of application and hinders the functional extensions. We prove that asymmetric searchable structure could be converted to symmetric structure, and functions could be modeled separately apart from the core searchable structure. Based on this observation, we propose a layered searchable encryption (LSE) scheme, which provides compatibility, flexibility, and security for various settings and functionalities. In this scheme, the outputs of the core searchable component based on either symmetric or asymmetric setting are converted to some uniform mappings, which are then transmitted to loosely coupled functional components to further filter the results. In such a way, all functional components could directly support both symmetric and asymmetric settings. Based on LSE, we propose two representative and novel constructions for ranked keyword query (previously only available in symmetric scheme) and range query (previously only available in asymmetric scheme).

## 1. Introduction

Cloud storage provides an elastic, highly available, easily accessible, and cheap repository to users to store and use their data, and such a convenient way attracts more and more people. In many cases, the users require their sensitive data, such as business documents, to be secure against any adversary or even the cloud provider, and therefore all data must be encrypted before sending to the server [1]. However, traditional encryption schemes (e.g., DES) do not provide any functionality to the users such that searching for the desired documents by keywords, as a basic function for storage system, is quite impossible. The problem is that there is no way to know if there exists such keywords in an encrypted document without decryption, and apparently the server should not have the decryption key.

Searchable encryption technique provides a solution to such problem. It enables the users to encrypt their sensitive data and store it to the remote server, while retaining the ability to search by keywords. While searching, the user sends to the server a secret token (a transformation of the queried keywords); then the server uses the token to search over the encrypted data and returns the matched documents. During the process, the server does not know what the queried keywords and the document contents are, and therefore the privacy is guaranteed.

Many searchable encryption schemes have been proposed with various settings and functionalities. For symmetric searchable encryption schemes, the user encrypts, searches, and decrypts the documents using his/her private symmetric key. For asymmetric searchable encryption schemes, the data sender encrypts the documents using the user's public key, and the user searches and decrypts the documents using the private key. Beyond the basic keyword matching, many functions are also added to either symmetric or asymmetric setting, such as range query, phrase query, and fuzzy keyword query.

However, these functions are often realized by different methods with different searchable structures which are generally not compatible with each other. For example, the asymmetric encryption scheme introduced in [2] realized conjunctive, subset, and range queries. However, it is difficult to figure out how to apply this method to symmetric setting. Even for the same setting, such as the fuzzy query scheme introduced in [3] and the rank-ordered query scheme introduced in [4], it is difficult to figure out how to combine two methods together since the functions are constructed based on different indexing structures.

Layered searchable encryption (LSE) scheme aims to provide compatibility, flexibility, and security for various settings and functionalities. In this new framework, keywords are firstly transformed to tokens that are filtered by the core searchable component (symmetric or asymmetric setting), and then the tokens are dynamically converted to uniform mappings which are transmitted to many stand-alone functional components (e.g., ranked keyword query component, fuzzy query component, etc.) to further filter the results. Since all functional components are independent of each other and the interfaces are common, the functions are compatible with each other and directly support both symmetric and asymmetric settings, and adding or deleting a function is quite simple since each function is loosely coupled with the core searchable component. Furthermore, LSE supports combined query. For example, the query “SELECT ∗ WHERE keywords = “cloud, storage, encryption” AND “security classification > 5” ORDERED BY “keyword:cloud”” (to express the query, we adopt the SQL-like format used in database) is a combination of three functional components: basic query, range query, and ranked keyword query (in this paper, we will present the concrete construction for this example).

Furthermore, this framework is similar to the data stream processing architecture [5], where functional components could be treated as operator boxes and the whole scheme could be treated as a data-flow system by which all processes follow the popular boxes and arrows paradigm. Therefore, in comparison to the previous searchable encryption schemes, LSE is more suitable for distributed and parallel computing environment.

In this paper, our contributions are the following. (1) We propose a novel framework for designing searchable encryption scheme called layered searchable encryption (LSE), which enables combined query and provides compatibility, flexibility, and security for various settings and functionalities. The new framework consists of a core searchable component with a symmetric/asymmetric converter, many functional components, and a common interface with new security model. (2) We propose a concrete construction for LSE that could theoretically combine all possible functionalities which are proposed in the recent years, and prove its semantic security for the interface. (3) As a complement for the prior works, we formally define two new security models for ranked keyword query and range query, called semantic security against chosen ranked keyword attack (CRKA) and chosen range attack (CRA) respectively, which provide integral security models for cryptographic analysis. (4) Based on LSE, we propose two representative and novel constructions for ranked keyword query component (previously only available in symmetric scheme) and range query component (previously only available in asymmetric scheme) and prove them semantically secure under the new security models.

The rest of the paper is organized as follows.  Section 2 presents the related work.  Section 3 presents the notations and preliminaries.  Section 4 presents the layered searchable encryption scheme and the concrete construction.  Section 5 discusses how to realize various functionalities and presents the concrete constructions for ranked keyword query and range query.  Section 6 concludes this paper.

## 2. Related Work

Searchable encryption schemes are designed to help the users to securely search over the encrypted data by keywords. The first scheme was introduced in [6] by Song et al., and later on many index-based symmetric searchable encryption (SSE) schemes were proposed. Goh introduced the first secure index in [7], and they also built the security model for searchable encryption called Adaptive Chosen Keyword Attack (IND-CKA). In [8], Curtmola et al. introduced two constructions to realize symmetric searchable encryption: the first construction (named SSE-1) is nonadaptive and the second one (named SSE-2) is adaptive. A generalization for symmetric searchable encryption was introduced in [9], and a representative SSE system designed by Microsoft was introduced in [10]. Another type of searchable encryption named asymmetric searchable encryption (ASE) is public-key based, which allows the user to search over the data encrypted by some data senders using the public key of the user. The first scheme was introduced in [11] by Boneh et al. based on bilinear maps, and the improved definition was introduced in [12].

There are many functional extensions for the searchable encryption schemes beyond the basic precise keyword matching. For symmetric setting, the authors in [4, 13, 14] introduced ranked keyword search schemes based on order-preserving encryption technique or two-round protocol, which allows the server to only return the top-*k* relevant results to the user. In [15], Golle et al. introduced a scheme supporting conjunctive keyword search which allows the user to search multiple keywords in a single query. In [3, 16, 17], the authors introduced fuzzy keyword search schemes based on wildcard technique, which allows the user to submit only part of the precise keyword. Similar to fuzzy keyword search but different, the authors in [18, 19] introduced similarity search schemes based on wildcard technique, which allows the server to return the results similar to the queried keyword. In [20, 21], the authors introduced phrase query schemes based on trusted client-side server or binary search, which allows the user to query a phrase instead of multiple independent keywords. For asymmetric setting, the authors in [22, 23] introduced range query schemes. In addition, Boneh et al. also introduced conjunctive and subset query in [22] based on bilinear maps.

Note that most of these techniques are not compatible with each other due to specific data structure and mathematical property. However, in the following sections, we will prove that functional structures and searchable structures could be separately constructed, and asymmetric structures could be converted to symmetric structures such that a compatible all-in-one scheme is possible.

## 3. Notations and Preliminaries

We write *x*←_*U*_ *X* to denote sampling element *x* uniformly random from a set *X* and write *x* ← *𝒜* to denote the output of an algorithm *𝒜*. We write *a*||*b* to denote the concatenation of two strings *a* and *b*. We write |*A*| to denote its cardinality if *A* is a set and write |*a*| to denote its bit length if *a* is a string. A function *μ*(*k*) : *ℕ* → ℝ is negligible, if for every positive polynomial *p*(·) there exists an inter *N* > 0 such that for all *k* > *N*, |*μ*(*k*)|<1/*p*(*k*). We write poly(*k*) and negl(*k*) to denote polynomial and negligible functions in *k*, respectively.

We write Δ = (*w*_1_,…, *w*_*n*_) to denote a dictionary of *n* words in lexicographic order. We assume that all words are of length polynomial in *k*. We write *d* to refer to a document that contains poly(*k*) words and write |*d*| to denote the size of the document in bytes. In some cases, we also write *d* to denote the document identifier that uniquely identifies the document, such as a memory location. We write X to denote a component or a scheme and write X.func(…) to denote the corresponding function for the component or an algorithm in the scheme.

## 4. Layered Searchable Encryption Scheme

Layered searchable encryption scheme aims to combine symmetric and asymmetric searchable encryption schemes to provide a uniform model for functional extensions. Therefore, we first revisit the basic symmetric and asymmetric searchable encryption models and then build the layered searchable encryption model based on these two different settings. After that, we introduce the security model of the new framework, and finally we present the concrete construction.

### 4.1. Revisiting Searchable Encryption

We adopt the definition introduced by Curtmola et al. in [8] as a representative model for symmetric searchable encryption scheme. In this setting, the user who searches for the documents is also the data sender who encrypts the documents. Therefore, some efficient searching techniques, such as using a global index, are used and the searchable structure may be a single index file for all stored documents. For consistency with other definitions, we make a little modification for the original definition, and define the scheme as follows.

**Definition 1 (symmetric searchable encryption)**

A symmetric searchable encryption (SSE) scheme is a collection of five polynomial-time algorithms SSE = (Gen, Enc, Token, Search, Dec) as follows.

  
*K*←Gen(1^*k*^) is a probabilistic algorithm that takes as input a security parameter *k* and outputs a secret key *K*. It is run by the user and the key is kept secret.
 (*γ*, *C*)←Enc(*K*, *D*) is a probabilistic algorithm that takes as input a secret key *K* and a document collection *D* = (*d*_1_,…, *d*_*n*_) and outputs a searchable structure *γ* and a sequence of encrypted documents *C* = (*c*_1_,…, *c*_*n*_). It enables a user to query some keywords and the server returns the matched documents. For instance, in an index-based symmetric searchable encryption scheme, *γ* is the secure index. It is run by the user and (*γ*, *C*) is sent to the server.
 
*t*←Token(*K*, *w*) is a deterministic algorithm that takes as input a secret key *K* and a keyword *w* and outputs a search token *t* (also named trapdoor or capacity). It is run by the user.
 
*C*′←Enc(*C*, *γ*, *t*) is a deterministic algorithm that takes as input the encrypted documents *C*, the searchable structure *γ*, and the search token *t* and outputs the matched documents (or identifiers) *C*′ = (*c*_1_,…, *c*_*m*_). It is run by the server and *C*′ is sent to the user.
 
*d*←Dec(*K*, *c*) is a deterministic algorithm that takes as input a secret key *K* and the encrypted document *c* and outputs the recovered plaintext *d*. It is run by the user.

We adopt the definition introduced by Boneh et al. in [11] as a representative model for asymmetric searchable encryption scheme. In this setting, the user generates the public key and the private key. The data sender encrypts the data using the public key, and the user searches and decrypts the data using the private key. The original definition only contains the searchable part, for consistency; we add two algorithms and define the asymmetric searchable encryption as follows.

**Definition 2 (asymmetric searchable encryption)**

An asymmetric searchable encryption (ASE) scheme is a collection of seven polynomial-time algorithms ASE = (Gen, PEKS, Enc, Token, Test, Search, Dec) as follows.

 
*K*←Gen(1^*k*^) is a probabilistic algorithm that takes as input a security parameter *k* and outputs a public/private key pair *K* = (*K*_pub_, *K*_priv_). It is run by the user and only *K*_priv_ is kept secret.
 
*s*←PEKS(*K*_pub_; *w*) is a probabilistic algorithm that takes as input a public key *K*_pub_ and a word *w* and outputs a searchable structure *s*. It is run by the data sender and *s* is attached to the encrypted message, and the combination is sent to the server.
 
*c*←Enc(*K*_pub_; *d*) is a probabilistic algorithm that takes as input a public key *K*_pub_ and a document (message) and outputs the ciphertext *c*. It is run by the data sender and *c* (followed by multiple searchable structures) is sent to the server.
 
*t*←Token(*K*_priv_; *w*) is a deterministic algorithm that takes as input a private key *K*_priv_ and a keyword *w* and outputs a search token *t*. It is run by the user.
 
*b*←Test(*K*_pub_; *s*; *t*) is a deterministic algorithm that takes as input the public key *K*_pub_, a searchable structure *s*←PEKS(*K*_pub_, *w*′), and a search token *t*←Token(*K*_priv_, *w*) and outputs *b* = 1 if *w* = *w*′ or *b* = 0 otherwise. It is run by the server.
 
*C*′←Search(*K*_pub_; *C*; *S*; *t*) is a deterministic algorithm that takes as input the public key *K*_pub_, the encrypted documents *C* = (*C*_1_,…, *C*_*n*_), the corresponding searchable structure set *S* = (*S*_1_,…, *S*_*n*_) (each *S*_*i*_ contains multiple searchable structures corresponding to the keywords of the document) and the search token *t* and outputs the matched documents *C*′ = (*c*_1_,…, *c*_*m*_) (the documents' searchable structures satisfying 1←Test(*K*_pub_, *s*, *t*)). It is run by the server and *C*′ is sent to the user.
 
*d*←Dec(*K*_priv_; *c*) is a probabilistic algorithm that takes as input a private key *K*_priv_ and a ciphertext *c* and outputs the plaintext *d*. It is run by the user.

Unlike symmetric setting, the definition of asymmetric setting only works on a single document. For a document collection, it does not make any difference since the user could execute the encryption algorithm for each document, respectively.

By comparing the definitions of the two different settings, there exists a common link between the queried keywords and the matched documents: the searchable structure which is constructed using either symmetric key or the public key. Note that the structure is probabilistic in the asymmetric setting, or else the server could directly launch the chosen plaintext attack using the public key. However, we say that for symmetric and asymmetric settings, the searchable structures are both run-time deterministic. To prove this property, we first introduce a lemma as follows.

**Lemma 3**

For asymmetric setting, if the token *t* generated using the private key *K*_*priv*_ is deterministic, then the searchable structure *s* encrypted using the public key *K*_*pub*_ is run-time deterministic when the the algorithm *ASE.Test* outputs 1, even if the encryption is probabilistic.

**Proof**

Recall that the algorithm *t*←Token(*K*_priv_, *w*) is deterministic and *s*←PEKS(*K*_pub_, *w*) is probabilistic. However, for a single document, there only exists a single *s* that links to *w*. When 1←Test(*K*_pub_, *s*, *t*), it implies that *t* matches *s*. We replace *t* with *s*; then the token *t* = *s*, which could be generated by the data sender, who could generate the token using the public key *t*←Token′(*K*_pub_, *w*) which is in fact the algorithm PEKS(*K*_pub_, *w*). It seems that the data sender has indirectly generated the token without having the private key. Therefore, when the output of the test is 1, both the token and the searchable structure map to *t*, which is deterministic.

Based on the lemma above, we introduce a theorem which guides us to construct the converter in the layered searchable encryption scheme.

**Theorem 4 (run-time invariance)**

For both symmetric and asymmetric settings, if the search token *t* is deterministic, then the searchable structure is run-time deterministic.

**Proof**

As proved in Lemma 3, the searchable structure is run-time deterministic for asymmetric setting. For symmetric setting, the searchable structure is encrypted using the symmetric key *γ*←Enc(*K*, *D*), which is probabilistic. The token *t*←Token(*K*, *w*) is deterministic. Similar to asymmetric setting, when executing the deterministic algorithm Search, the matched entries (probabilistic) map to *t*, and the mapping is deterministic (here, an entry is the encrypted data using symmetric encryption that contains the information about the matched document, such as the node in the inverted index [8]). In other words, the searchable structure is run-time deterministic because of the deterministic mapping.

### 4.2. Scheme Definition

**Figure 1:**
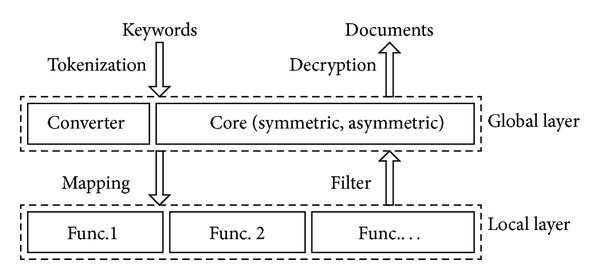
Architecture for layered searchable encryption scheme.

Our primary goal is to separate the functionalities from the searchable structures; therefore we consider to construct the basic searchable structures and various functions in different layers, as shown in Figure 1.

- Global layer: we name this layer “global” because all documents and all searchable structures are involved. In this layer, the basic searchable encryption scheme (symmetric or asymmetric) is executed and a global index could be constructed to improve search efficiency. The server receives the search tokens (each token is related to a keyword), executes the search procedure, and outputs the matched documents. Furthermore, the server converts the tokens (symmetric or asymmetric) to the corresponding mappings (another type of secret token) with uniform format and transfers the mappings with the matched documents (or identifiers) to the local layer.
- Local layer: we name this layer “local” because functional structures are constructed for each document independently. In this layer, each matched document is further filtered by all functions (e.g., phrase query function) which execute separately. Only the documents that pass all filter tests are returned to the global layer and finally return to the user.

For both layers, the framework consists of three different components: the core symmetric and asymmetric searchable components which provide basic keyword search, one or more functional components which provides various functionalities, and a converter. The converter is an algorithm that provides a uniform interface for both symmetric and asymmetric settings and provides uniform inputs for all functions. We note that all components in the two layers execute the search algorithm on the server side, and no trusted third-party is required. Now we formally define the scheme as follows.

**Definition 5 (layered searchable encryption)**

A layered searchable encryption (LSE) scheme is a collection of five polynomial-time algorithms LSE = (Gen, Enc, Token, Search, Dec) as follows.

 
*K*←Gen(1^*k*^) is a probabilistic algorithm that takes as input a security parameter *k* and outputs either a symmetric encryption key *K* = *K*_priv_ or an asymmetric encryption key pair *K* = (*K*_pub_, *K*_priv_). It is run by the user and only the public key *K*_pub_ is not kept secret.
 (*C*; *G*; *L*)←Enc(*K*_*e*_; *D*) is a probabilistic algorithm that takes as input an encryption key *K*_*e*_ (*K*_*e*_ = *K*_priv_ for symmetric setting or *K*_*e*_ = *K*_pub_ for asymmetric setting) and a document collection *D* = (*d*_1_,…, *d*_*n*_). It outputs *n* encrypted documents *C* = (*c*_1_,…, *c*_*n*_), a single (index-based) global searchable structure *G* or a sequence of global searchable structures *G* = (*G*_1_,…, *G*_*n*_) corresponding to *n* documents, and a sequence of local functional structures *L* = (*L*_1_,…, *L*_*n*_) corresponding to *n* encrypted documents. It is run by the data sender and (*C*, *G*, *L*) are sent to the server.
 
*T*←Token(*K*_priv_, *w*) is a deterministic algorithm that takes as input a secret key *K*_priv_ and a set of keywords *W* = (*w*_1_,…, *w*_*o*_) with functional instructions and outputs the corresponding search tokens *T* = (*t*_1_,…, *t*_*o*_) with functional instructions. It is run by the user and *T* is sent to the server.
 
*C*′←Search(*K*_pub_; *C*; *G*; *L*; *T*) is a deterministic algorithm that takes as input a public key *K*_pub_ (only for asymmetric setting), the encrypted documents *C*, the global searchable structure *G*, the local functional structure *L*, and the search token *T* and outputs the matched documents *C*′ = (*c*_1_,…, *c*_*m*_). It is run by the server and *C*′ is sent to the user.
 
*d*←Dec(*K*_priv_, *c*) is a deterministic algorithm that takes as input a secret key *K*_priv_ and an encrypted document *c*, and outputs the plaintext *d*. It is run by the user.

Functional instructions are separately specified by the functionalities and are written as a single SQL-like query. For example, the query “SELECT ∗ WHERE keywords = “cloud, storage, encryption” AND “security classification >5” ORDERED BY “keyword:cloud”” indicate that finding the documents that satisfying: containing the keywords “cloud, storage, encryption”, the security classification of the documents >5, sorting the matched documents by relevance score according to the keyword “cloud” and return the top-*k* relevant documents. Here we only write *W* = (*w*_1_,…, *w*_*o*_) (e.g., *W* = *“*cloud, storage, encryption”) as a representation for any instruction that contains the keywords. Similarly, the tokens *T* are just a representation for all functional instructions.

A functional component (FC) is a module in LSE that provides a specific functionality. It generates a local functional structure *L* for each encrypted document and provides filter service while searching. FC is designed to be compatible with both symmetric and asymmetric settings. Therefore, a conversion for the document as well as the query is required. We formally define the FC as follows.

**Definition 6 (functional component)**

A functional component (FC) is a collection of two polynomial-time algorithms FC = (Build, Filter) as follows.

 
*L*_*d*_←Build(*d*; *V*_*d*_) is an algorithm that takes as input a document *d* and the corresponding conversion *V*_*d*_ and outputs a functional structure *L*_*d*_. It is run by the data sender and *L*_*d*_ is appended to the encrypted document.
 
*C*′←Filter(*C*; *L*; *V*_*T*_) is an algorithm that takes as input the encrypted documents *C* = (*C*_1_,…, *C*_*x*_), the corresponding functional structure set *L* = (*L*_1_,…, *L*_*x*_), and the converted search tokens *V*_*T*_ = (*V*_1_,…, *V*_*x*_) and outputs a subset of documents *C*′. It is run by the server.

### 4.3. Security Model

The security of LSE relies on the algorithms used by the components. For example, if the symmetric searchable encryption scheme introduced in [8] is used as the core searchable component, then the core searchable structure guarantees that it is semantic secure against chosen keyword attack (CKA-secure). Similarly, the functional components have their individual security guarantees. Therefore, the whole LSE scheme does not have a uniform security model, and security models are built separately and each component could be analyzed independently. However, we could divide the security models into three parts: searchable component security, interface security, and functional component security. Searchable component security is guaranteed by the underlying core searchable encryption scheme. Therefore, we mainly discuss the other two security models.

The interface is common, and therefore the data that flow through the interface must be semantic secure. Informally speaking, it must guarantee that the adversary cannot distinguish the input and the output of each component from random strings. Semantic security against chosen plaintext attack (CPA) is very important for the interface, or else the security of some components will be correlated such that the loose coupling property is lost.

We first define the notion of plain trace, which is the direct information that could be captured from the data that flow through the interface.

**Definition 7 (plain trace)**

Let *D* = (*d*_1_,…, *d*_*n*_) be a document collection. Let *N* = (*N*_1_,…, *N*_*n*_) (only for asymmetric setting) be a keyword-counter set where *N*_*i*_ is the number of keywords in *d*_*i*_. Let the query history *W* = (*w*_1_,…, *w*_*p*_) be a sequence of queried keywords. Let the search pattern *σ*(*W*) be a *p* × *p* binary matrix such that for 1 ≤ *i*, *j* ≤ *p*, the *i*th row and *j*th column is 1 if *W*_*i*_ = *W*_*j*_ and 0 otherwise. The plain trace *π*(*D*, *W*) = (|*d*_1_ | ,…, |*d*_*n*_ | , *N*, *σ*(*W*)).

Note that plain trace is different from the notion of trace introduced in [8] which further captures the logic links. We will explain the reason after the definition of the security model. We now present the security model for the interface.

**Definition 8 (interface security against chosen plaintext attack, interface-CPA-secure)**

Let Σ be the layered searchable encryption scheme. Let *k* ∈ *ℕ* be the security parameter. We consider the following probabilistic experiments where *𝒜* is an adversary and *𝒮* is a simulator.

 Real_Σ,*𝒜*_(*k*): the challenger runs Gen(1^*k*^) to generate the key *K* = *K*_priv_ (symmetric) or *K* = (*K*_priv_, *K*_pub_) (asymmetric). The adversary *𝒜* generates a document collection *D* = (*d*_1_,…, *d*_*n*_), a sequence of query *W* = (*w*_1_,…, *w*_*p*_), and receives (*C*, *G*)←Enc(*K*_*e*_, *D*) and search tokens *T*←Token(*K*_priv_, *W*) from the challenger. *𝒜* generates a mapping *V*_*T*_ as the input for the functional component. Finally, *𝒜* returns a bit *b* that is output by the experiment.
 Sim_Σ,*𝒜*,*𝒮*_(*k*): given the plain trace *π*(*D*, *W*)*𝒮* generates (C*, G*) and T* and then sends the results to *𝒜*. *𝒜* generates a mapping *V*_T_* as the input for the functional component. Finally, *𝒜* returns a bit *b* that is output by the experiment.

We say that the interface of LSE is semantic secure against chosen plaintext attack, if for all PPT adversaries *𝒜*, there exists a PPT simulator *𝒮* such that

(1)   |Pr⁡[RealΣ,𝒜(k)=1]−Pr⁡[SimΣ,𝒜,𝒮(k)=1]|  ≤negl(k),

where the probabilities are over the coins of Gen.

Note that the functional structure *L* is not included here since the functional component is loosely coupled with the core. Therefore, the security of the functional component is separate from the framework and should be defined and analyzed separately.

The security model of the interface does not care about the search algorithm and the number of queries (therefore, only a single query sequence is presented). The reason is that the other information about the queried keywords and the documents are protected by the components. For example, if some documents are returned by one token, then the adversary could immediately infer that these documents have a common keyword (even the tokens and documents are indistinguishable from random in the interface), and such logic links could be hidden by generating multiple different tokens for one keyword (please refer to the adaptive construction in [8]) and the protection is guaranteed in the core searchable component.

Therefore, semantic security for the interface does not guarantee that the whole scheme is secure against chosen keyword attack or each component is secure under some other security models. However, it provides the basic security guarantee for the whole scheme and the independence for each component, and we will show such independence in the construction of the functional component later.

### 4.4. Concrete Construction

We first present the basic idea for the search process and the converter; then we present the template for constructing the functional component. Finally, we present the constructions for LSE (symmetric and asymmetric) in detail and prove the security of the interface.

#### 4.4.1. Basic Idea

**Figure 2:**
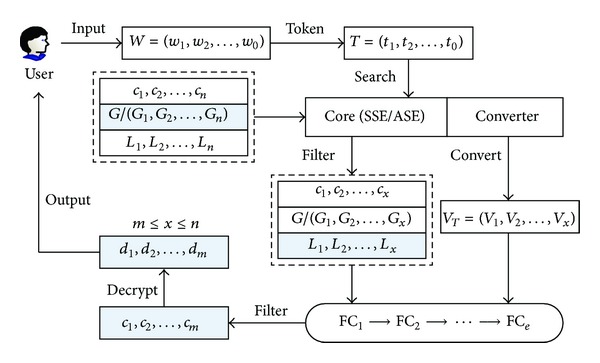
Search process of layered searchable encryption scheme.

As shown in Figure 2, the basic search process is as follows. The user transforms his queried keywords *W* to tokens *T* using the private key. The server receives the tokens *T* and executes the search procedure over all encrypted documents *c*_1_,…, *c*_*n*_. Each *c*_*i*_ (1 ≤ *i* ≤ *n*) is linked to a global searchable structure *G*_*i*_ (if a global index is used, then only a single searchable structure *G* is used for all encrypted documents) and a local functional structure *L*_*i*_, and only the global searchable structure *G*/(*G*_1_,…, *G*_*n*_) is used in this step. Then the tokens *T* are converted to the uniform tokens *V*_*T*_, and both *V*_*T*_ and the matched *x* encrypted documents are transmitted to functional components FC_1_,…, FC_*e*_ to further filter the results (e.g., phrase query filter). Each component outputs a subset of the input documents, and all components work serially since any document that does not pass the current filter will be unnecessary for the next filter. Finally, the matched encrypted documents *c*_1_,…, *c*_*m*_ are returned to the user and the user decrypts them to obtain the plaintexts *d*_1_,…, *d*_*m*_.

In order to construct a functional component that supports both symmetric and asymmetric settings, a conversion is needed to transform the plaintext to a kind of ciphertext that is independent from the settings. We call this independent ciphertext as a one-to-one “mapping” since each word in the plaintext has a deterministic token in the ciphertext. In addition, in order to provide a uniform format for the functional components, a hash function is used, and we will show the detailed construction in the next section. Now we present the template for the functional component (FC) in Algorithm 1.

We note that, in order to obtain the loose coupling property, any specific parameter is not allowed. Therefore, the uniform mappings of the words become the ideal common parameter. Another advantage of the mapping is that the main information needed for any functionality is retained: the difference of each word and the order of all words in the document. Based on this information, the word frequency, rank, subset, and so forth could also be inferred without the plaintext, which facilitates the designs of the Token and Search algorithms.

#### 4.4.2. Constructing Symmetric Part

For symmetric setting, the deterministic mapping of a document could be computed with ease. Let the tokens *t*_1_, *t*_2_, *t*_3_ map to the words “day,” “by,” and “night,” respectively. Then the deterministic mapping of a sentence could be written as

(2) “day  by  day,night  by  night”⟹t1t2t1t3t2t3.

Both the Enc and Token algorithms could generate these mappings, and the main process is as follows. For each document *d*, scan all words and compute the corresponding tokens, which are further hashed to the fixed-size mappings.

Suppose there are *n* documents *D* = (*d*_1_,…, *d*_*n*_), *n* corresponding ciphertexts *C* = (*c*_1_,…, *c*_*n*_), *e* functional components FC = (*F*_1_,…, *F*_*e*_), and *o* queried keywords *W* = (*w*_1_,…, *w*_*o*_). In addition, we define a hash function as follows:

(3) $$\begin{array}{c} f_{h}:{\left\{0,1\right\}}^{*}⟶{\left\{0,1\right\}}^{l}, \end{array}$$

where *l* is the length of the mapping according to the hash function. For example, if we use MD5 [24] as *f*_*h*_, then *l* is 128 bit. For clarity, we present the encryption scheme in Algorithm 2 and the search scheme in Algorithm 3 and finally present the complete scheme in Algorithm 4.

#### 4.4.3. Constructing Asymmetric Part

For asymmetric setting, the data sender does not have the private key; therefore the mapping will fail while searching since any encryption using the public key is probabilistic (CPA security). For example, let *e* represent an encryption of a word, and the same sentence will become (note that both *e*_1_ and *e*_3_ map to the word “day”)

(4) $$\begin{array}{c} “\text{day}\;\text{by}\;\text{day},\text{night}\;\text{by}\;\text{night”}\;⟹e_{1}e_{2}e_{3}e_{4}e_{5}e_{6}. \end{array}$$

Therefore, we delay the construction for such mapping after the construction of the searchable structure in algorithm Enc and use this searchable structure as an independent token for the corresponding word in algorithm Search. Recall that *s*←PEKS(*K*_pub_, *w*), then the tokens *t*_1_, *t*_2_, *t*_3_ which map to the words “day,” “by,” and “night” will be transformed to *s*_1_, *s*_2_, *s*_3_ when the test in the search algorithm outputs 1. Then we have

(5) $$\begin{array}{c} “\text{day}\;\text{by}\;\text{day},\text{night}\;\text{by}\;\text{night”}\;⟹s_{1}s_{2}s_{1}s_{3}s_{2}s_{3}. \end{array}$$

In this way, the data sender could construct the deterministic mapping for the document and indirectly obtain the deterministic tokens just using the public key. Similar to the symmetric setting, the process is as follows. For each document *d*, scan all words and compute the corresponding tokens according to searchable structures, which are further hashed to the fixed-size mappings. While searching, the tokens are mapped to different searchable structures according to each document.

**Figure 3:**
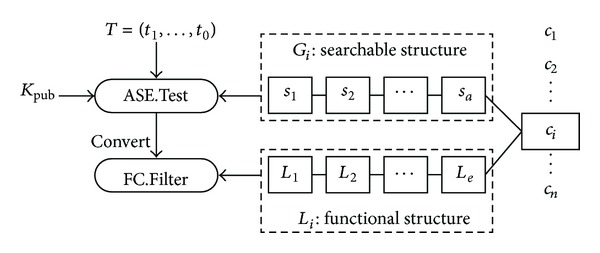
Data structure and search process for asymmetric setting.

There are some differences from the symmetric counterpart, as shown in Figure 3. First, the searchable structures are appended to each encrypted data such that the global index is not available. Second, a public key is involved for the searchable structure. However, due to the conversion, the public key is unnecessary for the functional components.

Now we present the encryption scheme in Algorithm 5 and the search scheme in Algorithm 6 and finally present the complete scheme in Algorithm 7.

We note that the process of “find *s*” at line 5 in Algorithm 6 could be simply done by directly using the intermediate results from the algorithm ASE.Search at line 1.

#### 4.4.4. Proof of Security

As we encapsulate the basic symmetric and asymmetric searchable encryptions in the global layer, the core is semantic secure against chosen keyword attack (CKA) [8, 9, 11]. The only thing we need is proving that the interface is CPA secure, and other functionalities are analyzed independently.

**Theorem 9**

If the core symmetric or asymmetric component is semantic secure against chosen keyword attack (CKA-secure), then LSE is interface-CPA-secure.

**Proof**

We briefly prove this theorem since the proof is straightforward. We claim that no polynomial-size distinguisher could distinguish (*C*, *G*, *T*, *V*_*T*_) from equal-size random strings (C*, G*, T*, *V*_T_*). As proved in [8, 11], the CKA-security of the core component guarantees that (*C*, *G*, *T*) are indistinguishable from (C*, G*, T*). For symmetric setting, *V*_*T*_ is the hash of *T* which is indistinguishable from T*. For asymmetric setting, *V*_*T*_ is the hash of the searchable structure *S* which is indistinguishable from random, say S*. Therefore, the hash value *V*_*T*_ is indistinguishable from the hash value *V*_T_*.

## 5. Realizing Various Functionalities

In this section, we show how to realize various functionalities based on LSE. We fist present the overview of the searchable encryption schemes with various functionalities and then propose two representative constructions for ranked keyword query and range query. Finally, we briefly discuss the methods for realizing the other functionalities.

### 5.1. Overview

As shown in Table 1, we present various functionalities for searchable encryption schemes: symmetric setting (Symm), asymmetric setting (Asym), ranked keyword query (Ranked keyword), range query (Range), phrase query (Phrase), fuzzy keyword query and wildcard query (Fuzzy keyword), similarity query (Similarity), and subset query (Subset). “Yes” means that the corresponding scheme directly supports such functionality. “Possible” means that the underlying data structure is compatible, and such functionality could be realized through minor modification of the original scheme. “—” means that realizing such functionality is quite challenging or the cost is relatively high.

### 5.2. Ranked Keyword Query

Ranked keyword query refers to a functionality that all matched documents are sorted according to some criteria, and only the top-*k*  relevant documents will be returned to the user. The SQL query format is “ORDERED BY ‘keyword'.” In [14], the authors introduced the computation for the relevance scores and proposed a comparing method over the encrypted scores based on order preserving symmetric encryption (OPSE) [27]. By using the same cryptographic primitive, the functional structure could record the encrypted relevance scores and setup an index with (token, score) pairs in order to obtain the score with *O*(1) computation complexity.

#### 5.2.1. Preliminaries

Order-preserving encryption (OPE) aims to encrypt the data in such a way that comparisons over the ciphertexts are possible. For *A*, *B*⊆*ℕ*, a function *f* : *A* → *B* is order-preserving if for all *i*, *j* ∈ *A*, *f*(*i*) < *f*(*j*) if and only if *i* < *j*. We say an encryption scheme OPE = (Enc, Dec) is order-preserving if Enc(*K*, ·) is an order-preserving function. In [28], Agrawal et al. proposed a representative OPE scheme that all numeric numbers are uniformly distributed. In [27], Boldyreva et al. introduced an order-preserving symmetric encryption scheme and proposed the security model. The improved definitions are introduced in [29]. Informally speaking, OPE is secure if the oracle access to OPE.Enc is indistinguishable from accessing to a random order-preserving function (ROPF). The security model is described as Pseudorandom Order-Preserving Function against Chosen Ciphertext Attack (POPF-CCA) [27].

A sparse look-up table is often managed by indirect addressing technique. Indirect addressing is also called FKS dictionary [30], which is used in symmetric searchable encryption scheme [8]. The addressing format is address, value, where the address is a virtual address that could locate the value field. Given the address, the algorithm will return the associated value in constant look-up time and return otherwise.

#### 5.2.2. Construction

We build a sparse look-up table  *A*  that records the pair (keyword, relevance score) with all data encrypted. When queried, the server searches the relevance scores of all documents and finds the top-*k* relevant documents. Note that, in order to security use OPE scheme to encrypt relevance scores, a preprocessing is necessary.

We build an OPE table to preprocess all plaintexts and store the encrypted relevance scores as follows. Given a document collection *D* = (*d*_1_,…, *d*_*n*_). For each document *d*_*k*_  (1 ≤ *k* ≤ *n*), scan it for *o* ^*k*^ keywords. Compute the relevance score (based on word frequency) *s*_*i*_^*k*^  (1 ≤ *i* ≤ *o* ^*k*^) for each keyword *w*_*i*_^*k*^ ∈ *W* in *d*_*k*_, and record a *o* ^*k*^ × 3 matrix for *d*_*k*_ with the *i*th line recording *R*_*i*_^*k*^ = (*w*_*i*_^*k*^, *s*_*i*_^*k*^, *p*_*i*_^*k*^), where *p*_*i*_^*k*^ is the position where the first *w*_*i*_^*k*^ occurs. For all documents, setup the OPE with *N* = *o* ^1^ + *o* ^2^ + ⋯+*o* ^*n*^ numbers (*s*_1_,…, *s*_*N*_). For each number *s*_*j*_  (1 ≤ *j* ≤ *N*), the encryption is *e*_*j*_. Transform the previous matrix to an OPE table with the *i*th line recording *R*_*i*_^*k*^ = (*w*_*i*_^*k*^, *e*_*i*_^*k*^, *p*_*i*_^*k*^) where *e*_*i*_^*k*^ is the encryption of *s*_*i*_^*k*^.

For a document, it has at most |*d* | /2 + 1 keywords (note that each keyword is followed by a separator such as a blank). The look-up table is padded to |*d* | /2 + 1 entries in order to achieve semantic security. Now we present the concrete construction for ranked keyword query component in Algorithm 8.

#### 5.2.3. Proof of Security

Informally speaking, the functional component must guarantee that given two documents' collection *D*_1_, *D*_2_ with equal size and |*D*_1_ | = |*D*_2_| then the challenger flips a coin *b* and encrypts *D*_*b*_ using LSE (the order of the ciphertexts are randomized). The adversary could query a keyword and receive the ordered document collection but he could not distinguish which one the challenger selected. By combining the security models defined in [8, 27], we formally define the notion of non-adaptive chosen ranked keyword attack (CRKA) as follows.

**Definition 10 (semantic security against nonadaptive chosen ranked keyword attack, CRKA-secure)**

Let Σ be the functional component for ranked keyword query. Let *k* ∈ *ℕ* be the security parameter. We consider the following probabilistic experiments, where *𝒜* is an adversary and *𝒮* is a simulator.

 Real_Σ,*𝒜*_(*k*): the challenger runs Gen(1^*k*^) to generate the key *K*. The adversary *𝒜* generates a document collection *D* = (*d*_1_,…, *d*_*n*_) (the size of each document is fixed) and receives the encrypted documents *C* = (*c*_1_,…, *c*_*n*_) and functional structures *L* = (*L*_1_,…, *L*_*n*_) with random order from the challenger. *𝒜* is allowed to query a keyword *w*, where *w* ∈ *d*_1_,…, *w* ∈ *d*_*n*_ and receives a mapping *v* from the challenger. Finally, *𝒜* returns a bit *b* that is output by the experiment.
 Sim_Σ,*𝒜*,*𝒮*_(*k*): given the number of documents *n*, the size of each document |*d*|, and the size of the mapping |*v*|, *𝒮* generates C*, L*, and v* and then sends the results to *𝒜*. Finally, *𝒜* returns a bit *b* that is output by the experiment.

We say that the functional component is CRKA-secure, if for all PPT adversaries *𝒜*, there exists a PPT simulator *𝒮* such that

(6)   |Pr[RealΣ,𝒜(k)=1]−Pr[SimΣ,𝒜,𝒮(k)=1]|  ≤negl(k),

where the probabilities are over the coins of Gen.

**Theorem 11**

If LSE is interface-CPA-secure and the underlying OPE is POPF-CCA secure, then the ranked keyword query component is CRKA-secure.

**Proof**

The simulator *𝒮* generates C*, L*, and v* as follows. As to C*, *𝒮* generates *n* random strings *c*_1_*,…, *c*_n_* of size |*d*|. As to L*, let *m* = |*d* | /2 + 1; *𝒮* generates *m* random strings V* = *v*_1_*,…, *v*_m_* with each has size |*v*|. *𝒮* generates an *m* × *n* matrix *E*_*m*×*n*_ = (*e*_ij_*), where each element *e*_ij_* is a random number. Then for each document, *𝒮* generates an index *A*_j_*[*v*_i_*] = *e*_ij_* (1 ≤ *i* ≤ *m*, 1 ≤ *j* ≤ *n*). As to v*, *𝒮* randomly selects v* = *v*_i_* ∈ V*.

We claim that no polynomial-size distinguisher could distinguish (*C*, *L*, *v*) from (C*, L*, v*). Since the encryption key *K* is kept secret from the adversary, the interface-CPA-security directly guarantees that C* is indistinguishable from *C*. It also guarantees that v* is indistinguishable from *v*. Upon receiving *v* = *v*_*i*_ ∈ *V* or v* = *v*_i_* ∈ V*, the adversary *𝒜* could invoke Filter(*C*, *L*, *v*) or Filter(C*, L*, v*) to obtain (*r*_1_ = *L*_1_[*v*_*i*_] = *e*_1_,…, *r*_*n*_ = *L*_*n*_[*v*_*i*_] = *e*_*n*_) or (*r*_1_* = *L*_1_*[*v*_i_*] = *e*_1_*,…, *r*_n_* = *L*_n_*[*v*_i_*] = *e*_n_*). POPF-CCA security guarantees that the set (*r*_1_,…, *r*_*n*_) is indistinguishable from (*r*_1_*,…, *r*_n_*); that is, the adversary is unable to distinguish the result of OPE from the result of a random order-preserving function. Therefore, *L* is indistinguishable from L*.

### 5.3. Range Query

Range query refers to a functionality that the server could test if the submitted keyword (integer) is within a range. The SQL query format is “WHERE ‘*x* operator *y*'.” For example, the user submits an integer *w*, and the server could return the documents where the corresponding searchable fields *a* satisfying *a* > *w*.

Although OPE could be applied here to support range query (similar to ranked keyword query), we propose another solution to demonstrate that how to apply the methods used in asymmetric setting to LSE. In [2], the authors introduced a construction based on bilinear map (asymmetric setting), which is not compatible with symmetric setting. However, the idea of transforming the comparison into a predicate (e.g., *P*_*a*_(*w*) = 1 if *a* > *w* where *P* is a predicate) could be used, and the functional structure could record all possible predicates and provide predicate test using a bloom filter.

#### 5.3.1. Preliminaries

A bloom filter [31] is a space-efficient probabilistic data structure that is used to test whether an element *s* is a member of a set *S* = (*s*_1_,…, *s*_*n*_). The set *S* is coded as an array *B* of *m* bits. Initially, all array bits are set to 0. The filter uses *r* independent hash functions *h*_1_,…, *h*_*r*_ where each *h*_*i*_ : {0,1}* → [1, *m*] for 1 ≤ *i* ≤ *r*. For each element *s*_*k*_ ∈ *S* where 1 ≤ *k* ≤ *n*, set the bits at positions *h*_1_(*s*_*k*_),…, *h*_*r*_(*s*_*k*_) to 1. Note that, a location could be set to 1 multiple times. To determine if *s* ∈ *S*, just check whether the positions *h*_1_(*s*),…, *h*_*r*_(*s*) in *B* are all 1. If any bit is 0, then *s* ∉ *S*. Otherwise, we say *s* ∈ *S* with high probability (the probability could be adjusted by parameters until acceptable).

In addition, we write $\bar{d}$ to denote the identifier of a document *d* such as the cryptographic hash of the pathname, and write *x* > (*y*_1_,…, *y*_*n*_) to denote *x* > *y*_1_,…, *x* > *y*_*n*_ for simplicity.

#### 5.3.2. Construction

For range query, the document *d* is labeled by some numbers. Here we only consider a single label *a*. Therefore, the aim of range query is to enable the user to submit a number *w* to search for the documents that satisfying the SQL-like query such as “WHERE “*a* > *w*””. We consider the five basic range query operators “>, ≥, <, ≤, = .” The other operators such as “*∈*” could be naturally derived from the basic operators.

We consider the whole range to be a sequence of *N* discrete numbers *A* = (*a*_1_,…, *a*_*N*_), where *a*_1_ < *a*_2_ < ⋯<*a*_*N*_. Then we set five shared virtual documents *d*_1_′ = (>*a*_1_, >*a*_2_,…, >*a*_*N*−1_), *d*_2_′ = (≥*a*_1_, ≥*a*_2_,…, ≥*a*_*N*_), *d*_3_′ = (<*a*_2_, <*a*_3_,…, <*a*_*N*_), *d*_4_′ = (≤*a*_1_, ≤*a*_2_,…, ≤*a*_*N*_), and *d*_5_′ = ( = *a*_1_, = *a*_2_,…, = *a*_*N*_) for all user's documents. The virtual document could be encrypted by LSE' core as a normal document. Therefore, for any keyword such as “>*a*_*i*_” where 1 ≤ *i* ≤ *N* − 1, there always exists a mapping *v*_*i*_.

Based on the notion of virtual document, a label *a* ∈ *A* for a user's document satisfying *a*_*i*−1_ < *a* < *a*_*i*+1_ (or *a* = *a*_*i*_) could be represented as 2*N* + 1 keywords *d* = (>*a*_1_, ≥*a*_1_,…, >*a*_*i*−1_, ≥*a*_*i*−1_, ≥*a*_*i*_, = *a*_*i*_, ≤*a*_*i*_, <*a*_*i*+1_, ≤*a*_*i*+1_,…, <*a*_*N*_, ≤*a*_*N*_), and these keywords are stored in a bloom filter *B*. Suppose the user queries a keyword “>*w*,” where *a*_1_ < *w* < *a*_*N*_; then the query is transmitted to the bloom filter to test if *“* > *w*” ∈*B*.

For example (we only consider the operator “>” here for simplicity), suppose we have two documents *c*_1_, *c*_2_ labeled 5,10, respectively. Then the transformed sets are *B*_1_ = (>1, >2,…, >4) and *B*_2_ = (>1, >2,…, >9). If the user submits >7, then only *B*_2_ matches the query, which is the same result as direct comparisons since 5≯7 and 10 > 7, and then *c*_2_ is returned. Similarly, the query “>3” will match both documents, and *c*_1_, *c*_2_ are returned.

Now we construct the secure version of the aforementioned scheme. Let *A* = (*a*_1_,…, *a*_*N*_) denote the domain of the label, and setup the bloom filter with *r* independent hash functions *h*_1_,…, *h*_*r*_. The identifier $\bar{d}$ of a document is always bound to the document *d* or the ciphertext *c*. The concrete construction is presented in Algorithm 9.

The size of the bloom filter could be dramatically reduced if the domain is bucketized [32] for example, bucketizing the subrange [10,20) as tag 10 and the subrange [20,30) as tag 20. Then a query for “>13” could be mapped to the closest query “>10.” In other words, the whole domain is divided to multiple subranges that the queried range is transformed to the approximate range. The optimization of the idea of bucketizing the range is introduced in [33]. In such way, the number of the data stored in the bloom filter will become smaller. However, this will induce inaccuracy for the query result.

#### 5.3.3. Proof of Security

For simplicity without loss of generality, we only consider the operator “>” here, and the other operators are the same. Informally speaking, the functional component must guarantee that the adversary is unable to guess the queried range as well as the range in the ciphertext, and the basic game works as follows. Given two documents *d*_1_, *d*_2_ that are labeled with two numbers *a*_1_, *a*_2_, respectively, the challenger flips a coin *b* and encrypts (*d*_*b*_, *a*_*b*_). The adversary is allowed to adaptively query *p* keywords *W* = (*w*_1_,…, *w*_*p*_), where each *w*_*i*_ ∈ *W* that (*a*_1_, *a*_2_) > *w*_*i*_. Note that querying *w*_*i*_ that *a*_1_ > *w*_*i*_,  *a*_2_≯*w*_*i*_ is not allowed since the document is immediately distinguished (only the document with *a*_1_ > *w*_*i*_ is matched and returned). We propose the notion of chosen range attack (CRA) and formally define the security model for semantic security as follows.

**Definition 12 (semantic security against chosen range attack, CRA-secure)**

Let Σ be the functional component for ranked keyword query. Let *k* ∈ *ℕ* be the security parameter. We consider the following probabilistic experiments, where *𝒜* is an adversary and *𝒮* is a simulator.

 Real_Σ,*𝒜*_(*k*): the challenger runs Gen(1^*k*^) to generate the key *K*. The adversary *𝒜* generates a document *d* and the labeled number *a* and receives the encrypted document *c* and the functional structure *L*. *𝒜* is allowed to adaptively query *p* keywords *W* = (>*w*_1_,…, >*w*_*p*_). For each query “>*w*_*i*_,” *𝒜* receives a mapping *v*_*i*_ from the challenger. Finally, *𝒜* returns a bit *b* that is output by the experiment.
 Sim_Σ,*𝒜*,*𝒮*_(*k*): given the document size |*d*|, the cardinality of the range *N*, and the size of the mapping |*v*|, *𝒮* generates c*, L*, and V* = (*v*_1_*,…, *v*_p_*), and then sends the results to *𝒜*. Finally, *𝒜* returns a bit *b* that is output by the experiment.

We say that the functional component is CRA-secure, if for all PPT adversaries *𝒜*, there exists a PPT simulator *𝒮* such that

(7) |Pr⁡[RealΣ,𝒜(k)=1]−Pr⁡[SimΣ,𝒜,𝒮(k)=1]|  ≤negl(k),

where the probabilities are over the coins of Gen.

**Theorem 13**

If LSE is interface-CPA-secure, then the ranked keyword query component is CRA-secure.

**Proof**

The simulator *𝒮* generates c*, L* and V* = (*v*_1_*,…, *v*_p_*) as follows. As to c*, *𝒮* generates a random string of size |*d*|. As to L*, *𝒮* generates a random string ${\bar{d}}^{*}$ and 2*N* + 1 distinct and random strings *T* = (*t*_1_,…, *t*_2*N*+1_). For each *t*_*i*_ ∈ *T*, *𝒮* computes *r* codewords $y_{1}^{*}=h_{1}({\bar{d}}^{*}||t_{i}),\ldots ,y_{r}^{*}=h_{r}({\bar{d}}^{*}||t_{i})$ and inserts the codewords *y*_1_*,…, *y*_r_* into a bloom filter B*. Let L* = B*. As to V*, for each *v*_i_* ∈ V*, *𝒮* randomly selects a distinct *t*_*j*_ ∈ *T* maps to *v*_i_*, such that *v*_i_* = *t*_*j*_. Note that, if *v*_x_* = *v*_y_* for some locations 1 ≤ *x*, *y* ≤ *p*, the mapping is the same.

We claim that no polynomial-size distinguisher could distinguish (*c*, *L*, *V*) from (c*, L*, V*). Since the encryption key *K* is kept secret from the adversary, the interface-CPA-security directly guarantees that c* is indistinguishable from *c*. It also guarantees that each *v*_*i*_ ∈ *V* is indistinguishable from the random string *v*_i_* such that *V* is indistinguishable from V*. Therefore, the locations (*y*_1_,…, *y*_*r*_) of *v*_*i*_ in bloom filter *B* is indistinguishable from the locations (*y*_1_*,…, *y*_r_*) of *v*_i_* in bloom filter B*. Therefore, *r* · (2*N* + 1) locations in *B* are indistinguishable from *r* · (2*N* + 1) locations in B*. Thus, *L* is indistinguishable from L*.

### 5.4. Other Functionalities

Due to space limitation, we only discuss the above two representative functional components. We briefly introduce how to realize some other functionalities based on LSE as follows.

*Phrase Query*. It refers to a query with consecutive and ordered multiple keywords. For example, searching with phrase “operating system” requires that not only each keyword “operating” and “system” must exist in each returned document, but also the order that “operating” is followed by “system” must also be satisfied. In [21], the authors introduced a solution based on Nextword Index [34]. It allows the index to record the keyword position for each document and enables the user to query the consecutive keywords based on binary search over all positions. However, it has *O*(log⁡*n*) computation complexity for each document. Based on LSE, this functionality could be realized using bloom filter (as demonstrated in range query scheme) which recording biword or more words based on Partial Phrase Indexes [35]. As a result, the scheme coude achieve approximately *O*(1) computation complexity (note that, the index in the global layer could reduce a large number of results for multiple keywords). 

*Fuzzy Keyword Search*. It refers to a functionality that the user submit a fragment of a keyword (or a keyword that does not exist in all documents) and the server could search for the documents with all possible keywords that are closed to the fragment. In [3], the authors introduced a wildcard-based construction that could handle fuzzy keyword search with arbitrary edit distance [36]. By using the same method, the functional structure could realize this functionality by recording and indexing the fuzzy set of all mappings instead of keywords. 

*Similarity Query*. It refers to a functionality that the server could return to the user some documents containing keywords which are similar to the queried keyword. In both [18, 19], the authors realized this functionality based on fuzzy set. Therefore, although different methods are used, the construction of the fundamental component is similar to the construction of fuzzy keyword search scheme. 

*Subset Query*. It refers to a functionality that the server could test if the queried message is a subset of the values in the searchable fields. For example, let *S* be a set that contains multiple e-mail addresses. If the user search for some encrypted mails containing Alice's e-mail *a*, then the server must have the ability to test if *a* ∈ *S* without knowing any other information. A solution was also introduced in [2]. Similar to the range query scheme, this test could also be viewed as a predicate and therefore the solution is the same.

### 5.5. Performance Analysis

**Figure 4:**
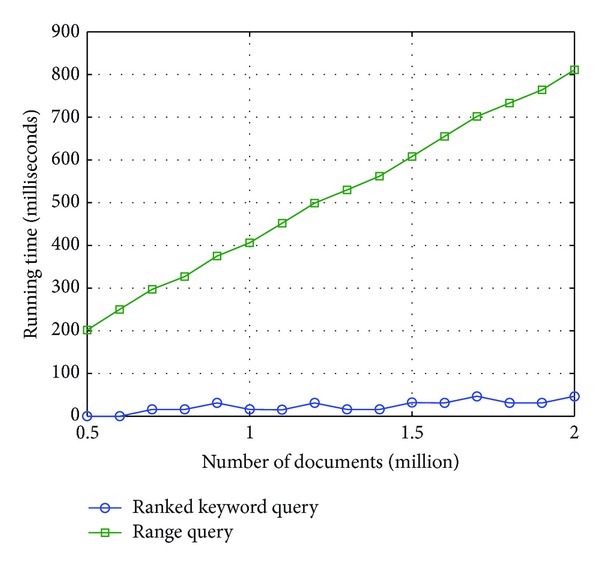
Time costs of the filter algorithms (single server, single query).

The algorithms of ranked keyword query component and range query component are coded in C++ programming language and the server is a Pentium Dual-Core E5300 PC with 2.6 GHz CPU. Each document is fixed to 10 KB with random words chosen from a dictionary, and the query is also some random keywords (random numbers). For bloom filter used in range query component, the number of hash functions is set to 8. The time costs of the filter algorithms are shown in Figure 4.

**Figure 5:**
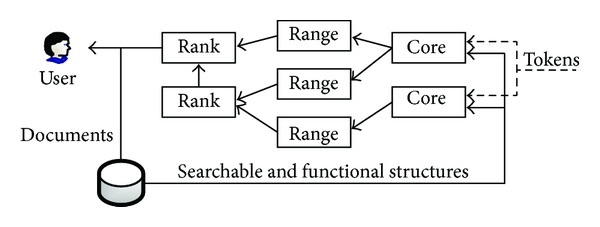
Deploying functional components to multiple servers.

Let *n* denote the number of documents. For ranked keyword query, the main operations are retrieving the relevance scores from the secure table managed by indirect addressing technique (*O*(*n*) search complexity) and selecting the top-*k* scores (*O*(*n*) computation complexity). For range query, the main operation is computing 8 hash values (*O*(*n*) computation complexity). Note that the current document will be passed if any position in bloom filter is 0. Therefore, not all eight hash functions are executed all the time. The figure demonstrates that, even for a single server, the algorithms are both efficient. Note that, since the functional components are loosely coupled with each other, they could be deployed to different servers. For example, two core components (Core), two ranked keyword query components (Rank), and three range query components (Range) could be executed as a data-flow boxes as shown in Figure 5. Each box could be deployed to any server. The detailed methods are out of scope of this paper and we will not discuss this further.

## 6. Conclusions

Layered searchable encryption scheme provides a new way of thinking the relationship among the searchable structure, functionality and security. It separates the functionalities apart from the core searchable structure without loss of security. Therefore, the loose coupling property provides compatibility for symmetric and asymmetric settings and it also provides flexibility for adding or deleting various functionalities. Furthermore, following the popular boxes and arrows paradigm, the loose coupling property makes the scheme more suitable for distributed and parallel computing environment.

## Figures and Tables

**Algorithm 1:**
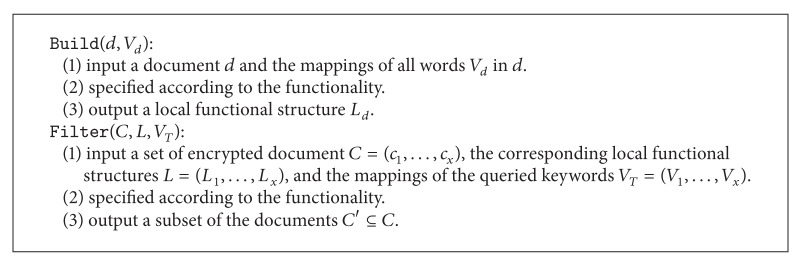
Template for functional component: F C.

**Algorithm 2:**
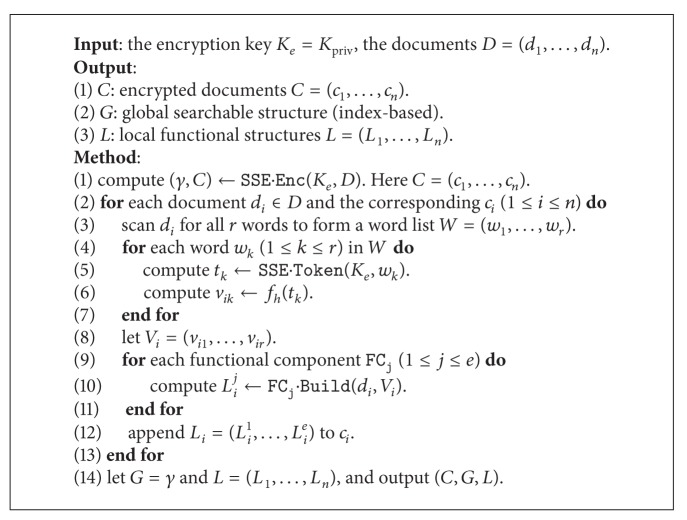
Encryption (symmetric): E nc(*K*_*e*_, *D*).

**Algorithm 3:**
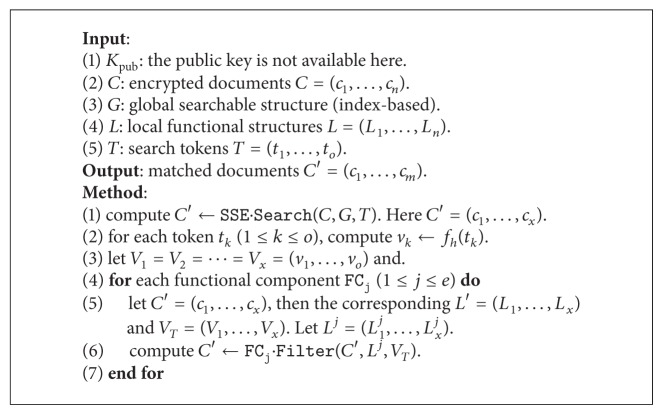
Search (symmetric): S earch(*K*_pub_, *C*, *G*, *L*, *T*).

**Algorithm 4:**
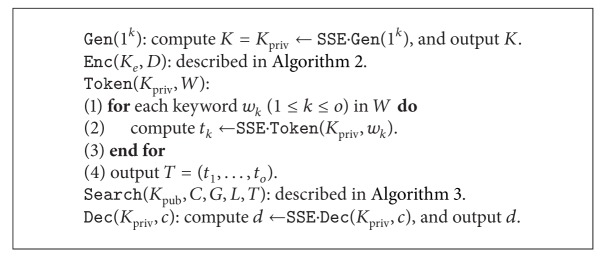
LSE scheme: symmetric part.

**Algorithm 5:**
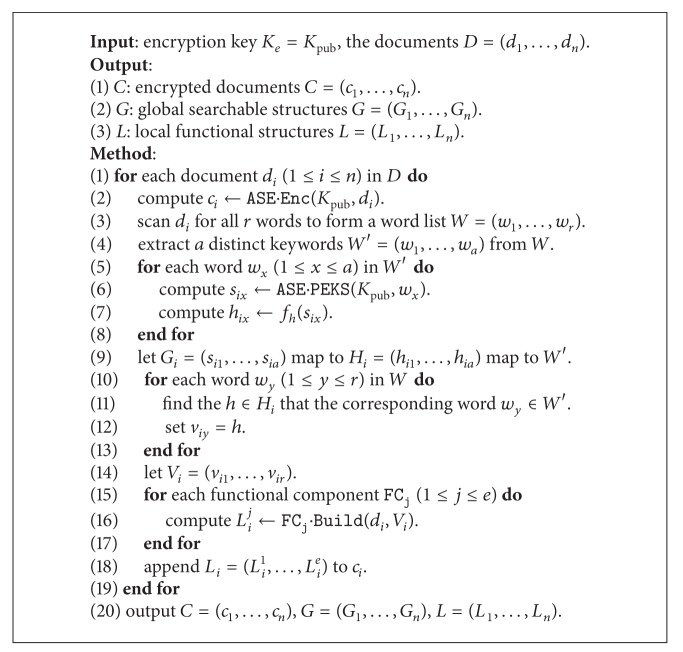
Encryption (asymmetric): E nc(*K*_*e*_, *D*).

**Algorithm 6:**
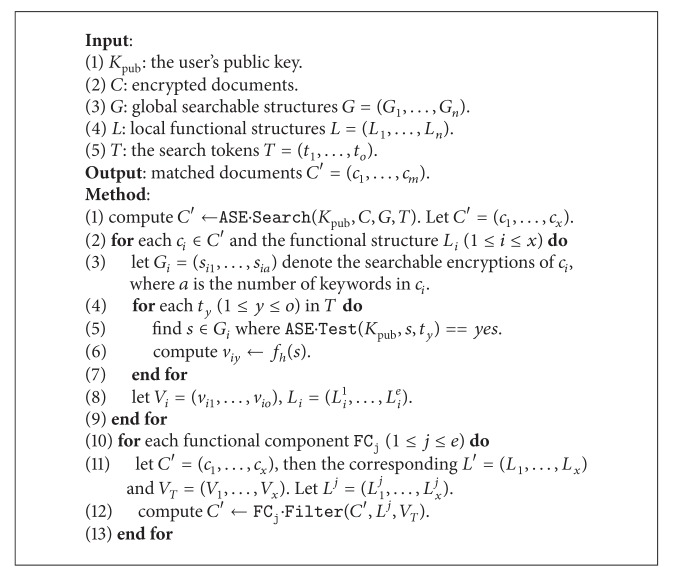
Search (asymmetric): S earch(*K*_pub_, *C*, *G*, *L*, *T*).

**Algorithm 7:**
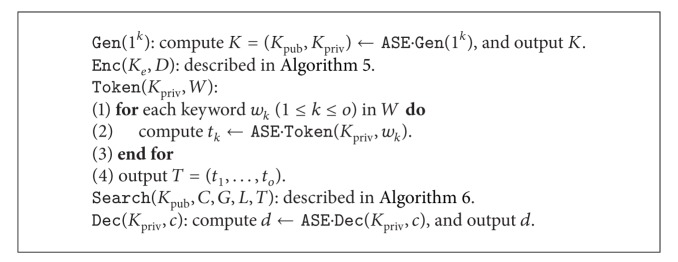
LSE scheme: asymmetric part.

**Algorithm 8:**
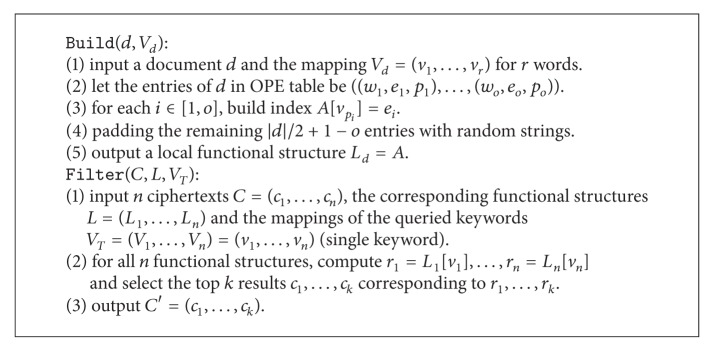
Ranked keyword query component.

**Algorithm 9:**
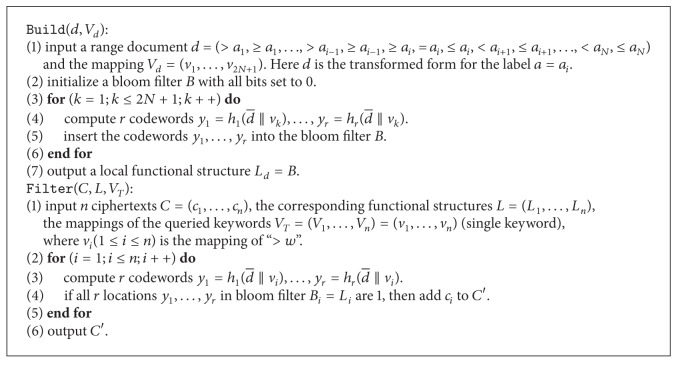
Range query component.

**Table 1:** Searchable encryption schemes with various functionalities.

	Symm.	Asymm.	Ranked keyword	Range	Phrase	Fuzzy keyword	Similarity	Subset
Ranked keyword query [4, 13, 14, 25]	Yes	—	Yes	—	Possible	Possible	Possible	—
Range query [22, 23]	—	Yes	—	Yes	—	Possible	Possible	Possible
Phrase query [20, 21]	Yes	—	Possible	—	Yes	Possible	Possible	—
Fuzzy keyword query [3, 16, 17]	Yes	—	Possible	—	Possible	Yes	Possible	—
Wildcard query [26]	—	Yes	—	Possible	—	Yes	Possible	Possible
Similarity query [18, 19]	Yes	—	Possible	—	Possible	Possible	Yes	—
Subset query [22]	—	Yes	—	Possible	—	Possible	Possible	Yes
This paper	Yes	Yes	Yes	Yes	Yes	Yes	Yes	Yes

## References

[1] Takabi H, Joshi JBD, Ahn G-J (2010). Security and privacy challenges in cloud computing environments. *IEEE Security and Privacy*.

[2] Boneh D, Waters B (2007). Conjunctive, subset, and range queries on encrypted data. *Theory of Cryptography*.

[3] Li J, Wang Q, Wang C, Cao N, Ren K, Lou W Fuzzy keyword search over encrypted data in cloud computing.

[4] Swaminathan A, Mao Y, Su G-M Confidentiality-preserving rank-ordered search.

[5] Abadi DJ, Carney D, Çetintemel U (2003). Aurora: a new model and architecture for data stream management. *VLDB Journal*.

[6] Song DX, Wagner D, Perrig A Practical techniques for searches on encrypted data.

[7] Goh EJ (2003). Secure indexes.

[8] Curtmola R, Garay J, Kamara S, Ostrovsky R Searchable symmetric encryption: improved definitions and efficient constructions.

[9] Chase M, Kamara S Structured encryption and controlled disclosure.

[10] Kamara S, Papamanthou C, Roeder T Cs2: a searchable cryptographic cloud storage system.

[11] Boneh D, Crescenzo GD, Ostrovsky R, Persiano G Public key encryption with keyword search.

[12] Abdalla M, Bellare M, Catalano D (2008). Searchable encryption revisited: consistency properties, relation to anonymous IBE, and extensions. *Journal of Cryptology*.

[13] Cao N, Wang C, Li M, Ren K, Lou W Privacy-preserving multi-keyword ranked search over encrypted cloud data.

[14] Wang C, Cao N, Li J, Ren K, Lou W Secure ranked keyword search over encrypted cloud data.

[15] Golle P, Staddon J, Waters B Secure conjunctive keyword search over encrypted data.

[16] Bosch C, Brinkman R, Hartel P, Jonker W Conjunctive wildcard search over encrypted data.

[17] Bringer J, Chabanne H Embedding edit distance to allow private keyword search in cloud computing.

[18] Cong W, Kui R, Shucheng Y, Urs KMR Achieving usable and privacy-assured similarity search over outsourced cloud data.

[19] Kuzu M, Islam MS, Kantarcioglu M Efficient similarity search over encrypted data.

[20] Zittrower S, Zou CC Encrypted phrase searching in the cloud.

[21] Tang Y, Gu D, Ding N, Lu H Phrase search over encrypted data with symmetric encryption scheme.

[22] Boneh D, Kushilevitz E, Ostrovsky R, Skeith WE Public key encryption that allows pir queries.

[23] Shi E, Bethencourt J, Chan T-HH, Song D, Perrig A Multi-dimensional range query over encrypted data.

[24] Rivest R (1992). *The Md5 Message-Digest Algorithm*.

[25] Wang C, Cao N, Ren K, Lou W (2012). Enabling secure and efficient ranked keyword search over outsourced cloud data. *IEEE Transactions on Parallel and Distributed Systems*.

[26] Sedghi S, Van Liesdonk P, Nikova S, Hartel P, Jonker W (2010). Searching keywords with wildcards on encrypted data. *Security and Cryptography For Networks*.

[27] Boldyreva A, Chenette N, Lee Y, Oneill A Order-preserving symmetric encryption.

[28] Agrawal R, Kiernan J, Srikant R, Xu Y Order preserving encryption for numeric data.

[29] Boldyreva A, Chenette N, O'Neill A Order-preserving encryption revisited: improved security analysis and alternative solutions.

[30] Fredman ML, Szemeredi E, Komlos J (1984). Storing a sparse table with o(1) worst case access time. *Journal of the ACM*.

[31] Bloom BH (1970). Space/time trade-offs in hash coding with allowable errors. *Communications of the ACM*.

[32] Hacigümüş H, Iyer B, Li C, Mehrotra S Executing SQL over encrypted data in the database-service-provider model.

[33] Hore B, Mehrotra S, Tsudik G A privacy-preserving index for range queries.

[34] Williams HE, Zobel J, Anderson P (1999). What's next? index structures for efficient phrase querying. *Australasian Database Conference*.

[35] Gutwin C, Paynter G, Witten I, Nevill-Manning C, Frank E (1999). Improving browsing in digital libraries with keyphrase indexes. *Decision Support Systems*.

[36] Levenstein V (1965). Binary codes capable of correcting spurious insertions and deletions of ones. *Problems of Information Transmission*.

